# The influence of acute exercise on bone biomarkers: protocol for a systematic review with meta-analysis

**DOI:** 10.1186/s13643-020-01551-y

**Published:** 2020-12-12

**Authors:** E. Dolan, A. Dumas, K. M. Keane, G. Bestetti, L. H. M. Freitas, B. Gualano, W. Kohrt, G. A. Kelley, R. M. R. Pereira, C. Sale, P. Swinton

**Affiliations:** 1 Applied Physiology and Nutrition Research Group, School of Physical Education and Sport; Faculdade de Medicina FMUSP, University of Sao Paulo, Sao Paulo, Brazil; 2 Department of Sport, Exercise and Rehabilitation, Northumbria University, Newcastle upon Tyne, UK; 3 Food Research Centre, University of São Paulo, Sao Paulo, SP Brazil; 4 Centre for Women’s Health Research, School of Medicine, University of Colorado, Aurora, USA; 5 Department of Biostatistics, West Virginia University, Morgantown, USA; 6 Bone Metabolism Laboratory, Rheumatology Division, Faculdade de Medicina FMUSP, Universidade de Sao Paulo, Sao Paulo, SP Brazil; 7 Musculoskeletal Physiology Research Group, Sport, Health and Performance Enhancement (SHAPE) Research Centre, School of Science and Technology, Nottingham Trent University, Nottingham, UK; 8 School of Health Sciences, Robert Gordon University, Aberdeen, UK

**Keywords:** Exercise, Bone, Meta-analysis, Systematic review

## Abstract

**Background:**

Bone is a plastic tissue that is responsive to its physical environment. As a result, exercise interventions represent a potential means to influence the bone. However, little is currently known about how various exercise and participant characteristics interact to influence bone metabolism. Acute, controlled, interventions provide an in vivo model through which the acute bone response to exercise can be investigated, typically by monitoring circulating bone biomarkers. Currently, substantial heterogeneity in factors such as study design, quality, exercise, and participant characteristics render it difficult to synthesize and evaluate the available evidence. Using a systematic review and meta-analytic approach, the aim of this investigation is to quantify the effect of an acute exercise bout on circulating bone biomarkers as well as examine the potential factors that may moderate this response, e.g., variation in participant, exercise, and sampling characteristics.

**Methods:**

This protocol was designed in accordance with the PRISMA-P guidelines. Seven databases (MEDLINE, Embase, Sport Discus, Cochrane CENTRAL, PEDro, LILACS, and Ibec) will be systematically searched and supplemented by a secondary screening of the reference lists of all included articles. The PICOS (Population, Intervention, Comparator, Outcomes and Study Design) approach was used to guide the determination of the eligibility criteria. Participants of any age, sex, training, or health status will be considered for inclusion. We will select studies that have measured the bone biomarker response before and after an acute exercise session. All biomarkers considered to represent the bone metabolism will be considered for inclusion, and sensitivity analyses will be conducted using reference biomarkers for the measurement of bone resorption and formation (namely β-CTX-1 and P1NP). Multi-level, meta-regression models within a Bayesian framework will be used to explore the main effect of acute exercise on bone biomarkers as well as potential moderating factors. The risk of bias for each individual study will be evaluated using a modified version of the Downs and Black checklist while certainty in resultant outcomes will be assessed using the Grading of Recommendations Assessment, Development and Evaluation (GRADE) approach.

**Discussion:**

A better understanding of the bone metabolic response to an acute bout of exercise has the potential to advance our understanding of the mechanisms through which this stimulus impacts bone metabolism, including factors that may moderate this response. Additionally, we will identify current gaps in the evidence base and provide recommendations to inform future research.

**Systematic review registration:**

This protocol was prospectively registered in the Open Science Framework Registry (https://osf.io/6f8dz)

**Supplementary Information:**

The online version contains supplementary material available at 10.1186/s13643-020-01551-y.

## Background

Bone is a plastic tissue that is responsive to its physical environment. As a result, exercise interventions have the potential to impact bone via a range of direct and indirect mechanisms [1–3]. These include the direct impact of physical activity-induced loading cycles [4], activity-specific metabolic signals such as alterations to calcium kinetics [5], redox balance [6], pH perturbations [7], and indirect signals mediated via other tissues, primarily skeletal muscle [8]. Substantial research and public resources are expended on investigating and implementing exercise-based interventions for populations who are susceptible to bone-related disorders. In general, activities that convey higher-impact, multi-directional, and unaccustomed loading patterns are considered to convey the greatest osteogenic stimulus, and for this reason, guidelines for the use of exercise to improve bone generally recommend that both resistance and impact-based modalities are employed [9–11]. This approach has been reported to be effective in many populations, with meta-analytic data reporting a positive effect of controlled exercise interventions on bone density in a range of populations that include pre- [12] and postmenopausal [13] women, older adults [14], individuals with osteoporosis [15], and children [16]. However, it is important to understand that several of the aforementioned meta-analyses included a number of studies that reported no effect of exercise on the bone, while some studies even suggest that the bone may be negatively impacted by very high volumes or intensities of exercise, e.g., in athletes competing in sports that emphasize leanness or those that rely upon repetitive loading cycles [17–20].

Currently, there is a lack of understanding regarding the mechanistic pathways through which the bone responds to exercise. As a result, the identification of what combination of participant and exercise characteristics determine whether an osteogenic, osteoneutral, or even an osteolytic effect will be induced remains elusive. Acute exercise interventions are commonly used as an in vivo model to investigate the bone response to exercise, and these types of interventions have much to contribute to advancing the understanding of the processes through which the bone responds to exercise (and other acute stimuli). Bone remodeling is the dominant process through which mature bone responds to exercise [21], and it comprises a sequential and synchronized process of bone activation, resorption, reversal, and formation [3, 22]. Circulating bone biomarkers, which are widely used in the clinical setting [23–25], are used to provide information on the dynamic state of bone remodeling. This is important because static indicators of bone health and function, such as bone mass measured by DXA, or microarchitecture as indicated by computed tomography or magnetic resonance imaging, are slow to respond to stimuli, with measurable changes taking months or even years to occur [26]. As a result, bone biomarkers which are capable of providing more immediate information on the current bone state are the only viable option available to evaluate the impact of controlled, acute, exercise interventions on bone metabolism and, thus, to help identify the pathways that influence this response.

Recently, our research group published a comprehensive narrative review; the intention of which was to synthesize and evaluate our current understanding of the bone metabolic response to exercise [21]. In that review, we observed that a single exercise session often elicits an increase in biomarkers that are indicative of bone resorption [27–30], indicating an initial catabolic response of bone to exercise. In contrast, longer-term adaptations to exercise training are often characterized by an increase in bone formation markers [31–39] that occur concurrently with positive changes in the bone mass or microarchitecture [36, 40–42]. These observations appear plausible when considered in the context of what is suggested about the bone remodeling cycle, whereby the initial osteoclastic activation may be required to trigger a subsequent increase in osteoblastic activity [22]. In order to further advance our understanding beyond these general observations, it is necessary to progress beyond dichotomous interpretations of increases/decreases/no changes in isolated bone biomarkers and instead consider the magnitude and context of the reported changes. However, this is challenging as considerable heterogeneity in research findings exists, most likely due to the large variation in the design, characteristics, and quality of available studies. Appropriate systematic review protocols and meta-analytic models are essential to overcome these challenges. However, to the best of our knowledge, no systematic review, with or without meta-analysis, has been conducted on this topic. Thus, the aim of this investigation is to use the systematic review and meta-analytic approach to quantify the effect of an acute bout of exercise on circulating biomarkers indicative of the bone metabolism, as well as investigate potential the factors that may moderate this response, e.g., variation in participant, exercise, and sampling characteristics.

## Methods

### Overview

The protocol for this review adheres to previously published guidelines [43] and includes all items described in the checklist of Preferred Reporting Items for Systematic Review and Meta-Analysis Protocols (PRISMA-P) [44]. The completed checklist is available in Supplementary File 1, and the protocol for this review was pre-registered in the Open Science Framework Registry (https://osf.io/6f8dz).

### Eligibility criteria

The PICOS (Population, Intervention, Comparator, Outcomes and Study Design) approach was used to guide the determination of the eligibility criteria for this review.

#### Population

In order to allow for the analysis of whether participant characteristics (sex, age, health or training status) will impact the bone biomarker response to a single bout of exercise, men and women of any age and health or training status will be considered for inclusion in this review.

#### Intervention

Studies that investigate a single exercise bout of any duration, intensity, or type will be considered for inclusion. This includes all interventions that require an active effort by the individual (including movement therapies such as yoga and tai chi), whereby passive interventions (e.g., vibration therapy) will be excluded. Exercise interventions will be categorized according to their type (e.g., resistance, aerobic, multi-modal, plyometric, calisthenics), duration (min), intensity (e.g., percentage of maximum capacity), total work (defined as duration × intensity), and impact level (i.e., high-impact/multi-directional; low-impact/repetitive; moderate-impact/repetitive; or low-impact with high muscular load). A more detailed explanation of these categories along with examples is provided in the codebook described in Supplementary File 2.

Given that bone biomarkers are acutely responsive to nutrient intake and status [45–47], a secondary analysis of studies that investigate how nutritional strategies influence the bone biomarker response to exercise will be conducted. For studies that investigate exercise interventions with and without a specific nutritional intervention (e.g., calcium [27] or carbohydrate [48] supplementation), data from the non-nutritional condition will be used in the main meta-analysis while the nutritionally manipulated condition will be used to inform this secondary analysis. In addition, whether the exercise bout is conducted in a fed or fasted state will be considered.

#### Comparator

The primary comparison of interest is the pre-post difference in the bone biomarkers as a result of an acute exercise bout. Estimation of typical error in the bone biomarker assessments across the different time periods will be assessed using data from any of the eligible studies that include a non-exercise control condition as well as available data on circadian variation in bone biomarkers [47, 49]. This data will be accounted for within the meta-analytic model for the purpose of providing a more precise estimate of the true effect of exercise on the bone. In addition, sensitivity analysis limited to those studies that report data for a non-exercise control group will be conducted and compared to the results of the main model to identify whether the exclusion of a non-exercise control group, as is common in these types of studies, meaningfully influences effect size estimation.

#### Outcomes

The primary outcome of interest is the bone biomarker response to an acute exercise bout. Bone biomarkers will be considered in relation to the processes of (1) *bone formation* (bone-specific alkaline phosphatase (B-ALP), dickkopf-1 (DKK-1), carboxyterminal propeptide of type 1 procollagen (P1CP), N-terminal propeptide of type 1 procollagen (P1NP), and sclerostin), (2) *bone resorption* (pyridinoline (Pyr), deoxypyridinoline (Dpd), carboxyterminal telopeptide of type 1 procollagen (ICTP), aminoterminal telopeptide of type 1 collagen (NTx), cathepsin K, C-terminal telopeptide of type 1 collagen (β-CTX-1), tartrate resistance acid phosphatase isoenzyme 5b (TRAP5b), ratio of osteoprotegerin to receptor activator NF kappaB ligand (OPG/RANKL), hydroxylysine, and hydroxyproline), and (3) general *bone remodeling* (osteopontin and total and undercarboxylated osteocalcin (T/U-OC)). These are all commonly reported biomarkers that have been suggested to measure the processes of bone metabolism, although some are non-specific to the bone and/or are difficult to accurately measure [21]. For this reason, we will conduct sensitivity analyses using only those markers that have been designated as reference markers for the assessment of bone formation (P1NP) and resorption (β-CTX-1). These particular biomarkers were chosen based upon their relatively high specificity to the bone metabolism, their relatively small biological variability, and their responsiveness to osteogenic intervention [23–25, 50]. An additional sensitivity analysis will also be undertaken using all bone biomarkers apart from B-ALP, as it is suggested to represent late-stage bone mineralization and, as such, is unlikely to respond to a single exercise bout. Biomarkers indicative of calcium metabolism (circulating ionized or albumin-adjusted calcium, phosphorus, and parathyroid hormone) will be considered a secondary outcome. In the case of multiple available outcomes due to repeated sampling, the pre-exercise sample that is taken closest to the start of the exercise bout will be selected as the baseline value, while all sampling points during and post-exercise will be extracted and used to model the time course of the observed bone biomarker changes.

Fixed effects related to the study, participants, sampling, and exercise characteristics will be incorporated into the model to identify potential factors that may influence the main effect of acute exercise on bone biomarkers. These include (1) participant characteristics (age, sex, training status, health status), (2) exercise characteristics (type, duration, intensity, total work done, impact level), and (3) blood sampling characteristics (nutritional status, assay type, sample timing). A more detailed description of all data that will be extracted is described in the accompanying codebook (Supplementary File 2).

#### Outcome prioritization

As described in our recent narrative review, it appears that total work performed during the exercise bout, i.e., higher intensity efforts conducted for longer periods of time, may have a greater influence on the bone biomarker response than either intensity or duration alone [21]. Therefore, we hypothesize that this factor will be the most influential moderator of the main effect. In contrast to the commonly held view that impact is required to elicit an osteogenic response [2], it appears that low-impact, repetitive loading exercises such as cycling regularly elicit a bone biomarker response [5, 27, 51], indicating that exercise type and impact level may not exert a strong influence on the main effect. In relation to participant characteristics, age is likely to be an important factor determining the bone biomarker response to exercise given that children and young adults are thought to have greater bone plasticity, while older adults may experience age-related osteogenic resistance [52]. It is unlikely that sufficient data will be available to investigate the independent influence of each of these stated factors, e.g., most of the studies conducted to date have used young, healthy, men. As a result, there may be insufficient data on older adults, or women, to allow for the analysis of the moderating influence of these factors. When insufficient data is available for the analysis (defined as a minimum of four data points per group for categorical variables or 10 data points for continuous variables [53]), these will be suggested as potential directions for future research.

#### Study design

Any study design that includes measurement of bone biomarkers before and after an acute bout of exercise will be considered for inclusion. These include randomized and non-randomized controlled trials, including cross-over trials, as well as single group pre-post studies. Our rationale for including these different designs is based on (1) our desire to be as inclusive as possible, (2) to identify if the results are associated with these different designs, and (3) provide direction for future research with respect to designing studies aimed at examining the effects of an acute bout of exercise on bone biomarkers. Natural experiments, i.e., studies that measure bone biomarkers before and after real-life athletic events (e.g., endurance events) will also be considered for inclusion; however, because these investigations are prone to substantially more variation than experimental laboratory studies, they will not be included in the main analysis, and instead, a secondary analysis will be undertaken using these data.

### Information sources

Seven electronic databases, namely MEDLINE, Embase, Cochrante CENTRAL, Sport Discus, PEDro, LILACS, and IBEC will be used to source material for this review. MEDLINE, Embase, and Cochrane CENTRAL were selected based on the recommendations of the Cochrane Collaboration Handbook for Systematic Reviews of Interventions [54]. MEDLINE and Embase will be accessed using the OVID platform. Sport Discus and PEDro will be searched based on their specific relevance for this topic area, while the LILACS and IBECS databases will be accessed via the Virtual Health Library portal in order to identify Latin American- and Caribbean-based literature as well as health science journals published in Spanish. The primary database search strategy will be supplemented by citation screening of all studies included in the review along with relevant reviews and book chapters (e.g., Banfi et al. [55], Dolan et al. [21], and Alp [56]).

### Search strategy

All searches will be conducted by ED. Free-text terms related to each of the core concepts to be explored in this review will be used in each of the aforementioned databases. These include bone AND (exercise OR physical activity) AND (biomarkers OR turnover OR remodeling OR formation OR resorption). A combination of free-text and database-specific subject headings will be used to more comprehensively assess all available studies. The MEDLINE and Embase searches will be conducted using the OVID platform, and the advanced search multi-purpose (mp) function will be used. This function simultaneously searches several fields, including mapping free-text terms to the relevant database-specific subject headings, namely MeSH headings for MEDLINE and Emtree for Embase. Searches will be limited to human studies, and no restrictions will be placed on either date or language. Our research team comprises native English, Portuguese, and Spanish speakers. Eligible studies in any other language will be translated into English using feely available online translators (Google Translate or Babelfish). If a potentially eligible article cannot be adequately translated, we will contact the study authors for clarification, and if no response is obtained the article will be excluded from the review. Only peer-review studies published in scientific journals will be considered for inclusion in this review. Unpublished work, defined as master’s theses, dissertations, abstracts from conference proceedings, and technical reports will not be included. Our rationale is based on the work of van Driel et al. [57] who concluded that (1) the difficulty in retrieving unpublished work could lead to selection bias, (2) many unpublished trials are eventually published, (3) the methodological quality of such studies is poorer than those that are published, and (4) the effort and resources required to obtain unpublished work may not be warranted. In line with Cochrane Collaboration recommendations [54], the full search strategy for the MEDLINE search was submitted for peer review to an information scientist using the Peer Review for Electronic Search Strategy (PRESS) Guideline Assessment form [58] and is available in Supplementary File 3. This search strategy will be replicated for each of the other databases, and the individual search strategies will be reported as a supplementary file to the final manuscript. Search results from each database will be downloaded as a .ris file then uploaded to a systematic review management software (covidence.org) and deduplicated using the automatic option provided therein. In the case that any duplicate records are not detected using this automatic option, they will be manually removed during the screening process.

### Study records

#### Study selection

**Fig. 1:**
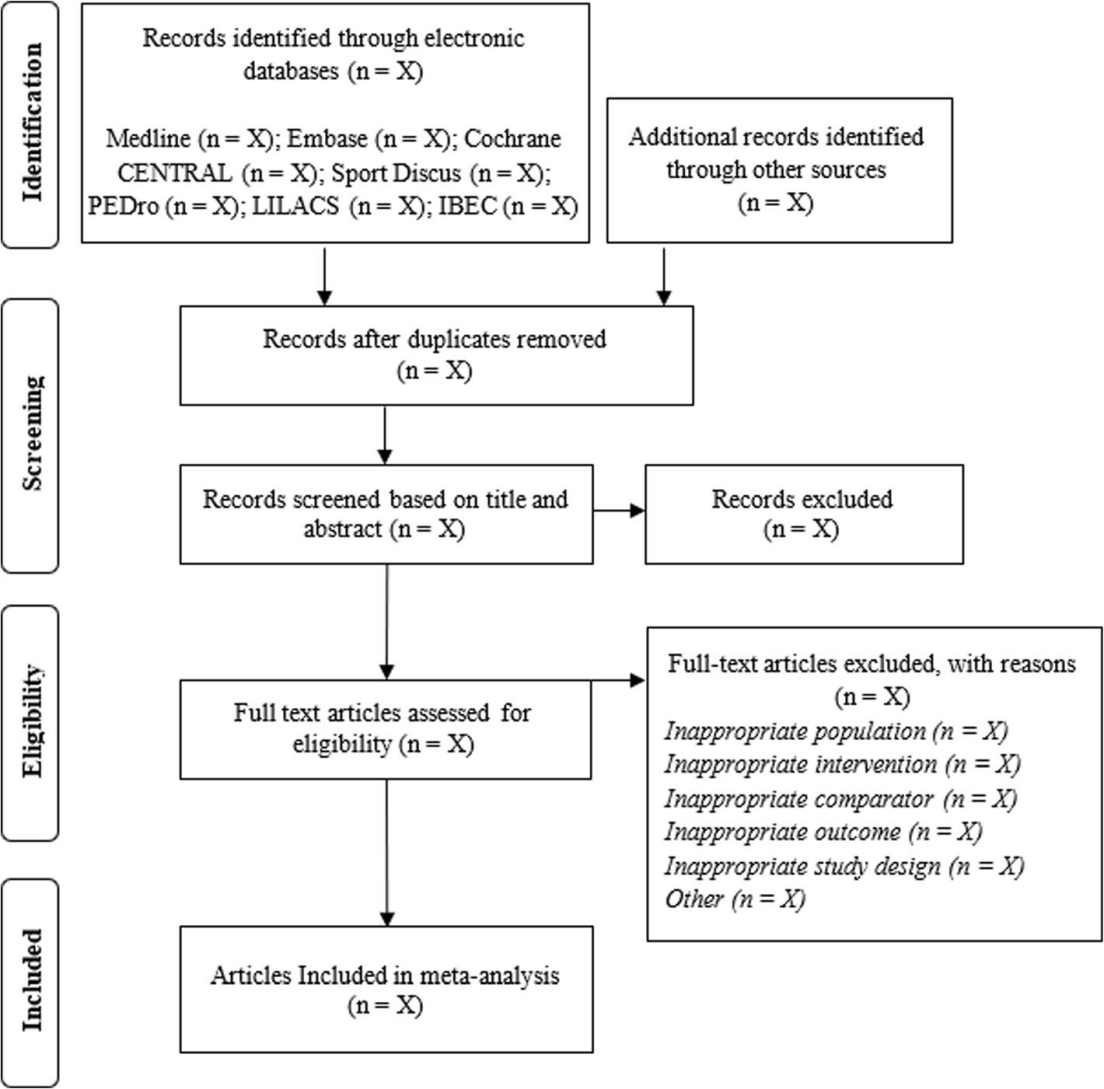
PRISMA flow diagram depicting the search process

A three-stage selection strategy will be independently undertaken by two members of the review team (KK and ED; title/abstract screen; full-text screen/full-text appraisal), and the results will be filtered using the eligibility criteria described above. The independent screeners will not be blinded to any study information and will convene at the end of each screening stage to resolve any discrepancies. These discrepancies will be resolved by a discussion, with a third party invited to mediate if required. During the full-text screen and review stages, reasons for exclusion will be categorized as one or more of the following: (1) inappropriate population, (2) inappropriate intervention, (3) inappropriate comparator, (4) inappropriate outcome, (5) inappropriate study design, and (6) others. The search strategy will be schematically illustrated using the PRISMA search flow diagram (see Fig. 1).

#### Data extraction

Data will be independently extracted by 2 members of the review team (AD/LHMF and ED/GB) into 2 independent pre-piloted excel sheets. All data will be independently coded by AD and ED as described in the accompanying codebook (Supplementary File 3). Prior to compiling all data into a single master sheet, AD and ED will meet to review all selections for agreement. Discrepancies will be resolved by a consensus, and if consensus cannot be reached, a third member of the review team will be invited to mediate. Prior to correcting disagreements, the overall agreement rate will be calculated using the Cohens *K* statistic [59]. If all required data is not available in the published article, study authors will be contacted by ED to request additional information (maximum of two e-mail attempts). If the primary outcome data is not available (either from the original paper or on request from the authors), then the paper will be excluded from the study. Our hierarchical random effects model means that results from studies can be included even if some secondary data is missing, and so studies will only be excluded if the primary data is not available, i.e., data on bone biomarkers recorded before and after an acute exercise session.

### Risk of bias assessment in individual studies

The risk of bias for each individual study will be independently assessed in duplicate by KK and AD/ED using a modified version of the Downs & Black checklist [50]. This tool was chosen over others as it provides a comprehensive assessment of the methodological quality of both randomized and non-randomized trials in healthcare research and has been validated as a tool to ascertain quality of reporting as well as internal and external validity [60]. Some items in the original tool were deemed unnecessary for this review, either because they were specifically relevant to longitudinal interventions and therefore not required in an investigation on the biomarker response to an acute exercise bout or because they related to the quality of reporting on factors that were not deemed to potentially bias the specific outcomes of interest in this review. The modified tool is available in Supplementary File 4. The results of this assessment will not be used to exclude any eligible studies. Rather, it will contribute to ascertaining confidence in the cumulative evidence of obtained results as well as highlight specific areas that future investigations on this topic should address to improve the quality of future research.

### Data synthesis

A Bayesian framework was chosen over a frequentist approach as it provides a more flexible modeling approach that will enable results to be interpreted intuitively through reporting of subjective probabilities [61]. The effect of exercise on bone biomarkers will be quantified by the effect sizes calculated with standardized mean differences pre- and post-exercise. Three-level random-effects Bayesian hierarchical models will be used to pool effect sizes and model average effects, variance within studies, variance between studies, and covariance of multiple outcomes reported in the same study (e.g., multiple bone biomarkers and/or single bone biomarker reported at multiple post-exercise time points). Within-study variance is influenced by pre-post correlations [62] that are generally not reported. Primary data obtained from relevant studies (including that produced in the laboratories of the study team) will be used to develop informative priors to model these within-study variances. Non-informative priors will be used for all other model parameters. Inconsistency in models will be described by comparing variances across the three levels. Inferences from all analyses will be performed on posterior samples generated using the Hamiltonian Markov Chain Monte Carlo method and through the use of credible intervals and calculated probabilities. Interpretations will be based on the range of values within the credible intervals and calculation of probabilities that the magnitude of the average effect size exceeds commonly used qualitative thresholds (e.g., small (0.2), medium (0.5), large (0.8) [63]). Analyses will be performed using the R wrapper package *brms* interfaced with *Stan* to perform the sampling [64]. The primary meta-analyses will comprise three univariate models with outcomes categorized as (1) bone formation, (2) bone resorption, and (3) bone remodeling. Sensitivity analyses will be conducted with restriction to reference markers for bone formation and resorption (P1NP and β-CTX-1). When possible, meta-regression will be used to explore the effect of various potential moderators as described above in the “Outcomes” section. Meta-regression will be performed when there is sufficient data including a minimum of four data points per category level or 10 data points for continuous variables [53]. Small-study effects (publication bias, etc.) will be visually inspected with funnel plots and quantified with a multi-level extension of Egger’s regression-intercept test [65].

### Confidence in cumulative evidence

The strength of evidence will be independently assessed in duplicate by ED and AD using the Grading of Recommendations Assessment Development and Evaluation (GRADE) instrument [66]. Potential downgrading factors include risk of bias, inconsistency, indirectness, imprecision, or the presence of publication bias. The risk of bias will be assessed using a modified Downs and Black checklist as described above, with the median assessment used to describe the resultant outcomes. In relation to the directness of outcomes, any data point that does not include a non-exercise control group will be downgraded a level. Median ratings from the risk of bias and indirectness assessments will be used to cumulatively describe these assessments for each outcome described in the review. Consistency will be ascertained using the meta-analysis results, and based on visual inspection of the effect size estimates, whether or not confidence intervals overlap, and on statistical tests for heterogeneity. Precision will be judged based on the number of outcomes available and on visual analysis of the width of the confidence intervals. Small-study effects (publication bias, etc.), will be assessed using Egger’s regression-intercept test along with visual inspection of funnel plots. Potential upgrading factors included the presence of large effects, evidence of dose-response, and the presence of plausible residual confounding factors.

## Conclusions

A better understanding of the bone metabolic response to acute exercise has the potential to advance our understanding of the mechanisms through which this stimulus impacts bone metabolism. The comprehensiveness of the review will also allow identification of current gaps in the evidence base and subsequent recommendations to inform future research. Broadly, these recommendations will include the identification of pertinent research questions that are currently under-studied as well as the highlighting of methodological issues that were apparent across the evidence base as a whole, and which should be corrected to improve future research efforts. For example, although this analysis will consider all biomarkers that are suggested to effect bone metabolism, many of these are non-specific to the bone and/or are difficult to measure. As a result, we expect that the current analysis will provide insight as to which of these markers are most likely to effect exercise-induced changes in bone metabolism and, thus, will help to guide future research in this area. Finally, the results of this investigation will be disseminated via a presentation at relevant conferences and publications in peer-reviewed journals, serving as a contemporary foundation on which on-going research efforts in this topic can be based.

## Supplementary Information

**Additional file 1.** PRISMA-P 2015 Checklist.

**Additional file 2.** Codebook.

**Additional file 3.** PRESS Guideline — Search Submission & Peer Review Assessment.

**Additional file 4.** Modified Downs & Black checklist.

## Data Availability

All data generated or analyzed during this study will be included in the published article and its supplementary files. Should any additional information be required, it will be made available from the corresponding author on reasonable request.

## Abbreviations

BMU: Basic multicellular unit
DXA: Dual-energy X-ray absorptiometry
PRISMA-P: Preferred Reporting Items for Systematic Review and Meta-Analysis Protocols
OSF: Open Science Framework
PICOS: Population, Intervention, Comparator, Outcomes and Study Design
B-ALP: Bone-specific alkaline phosphatase
DKK-1: Dickkopf-1
P1CP: Carboxyterminal propeptide of type 1 procollagen
P1NP: N-terminal propeptide of type 1 procollagen
Pyr: Pyridinoline
Dpd: Deoxypyridinoline
ICTP: Carboxyterminal telopeptide of type 1 procollagen
NTx: Aminoterminal telopeptide of type 1 collagen
β-CTX-1: C-terminal telopeptide of type 1 collagen
TRAP5b: Tartrate resistance acid phosphatase isoenzyme 5b
OPG: Osteoprotegerin
RANKL: Receptor activator NF kappaB ligand
T/U-OC: Total and undercarboxylated osteocalcin
RIS: Research information systems
BMI: Body mass index
GRADE: Grading of Recommendations Assessment Development and Evaluation
