# Supplementary material for: The influence of acute exercise on bone biomarkers: protocol for a systematic review with meta-analysis
Source: Syst Rev. 2020 Dec 12;9:291. doi: 10.1186/s13643-020-01551-y (PMC7733242; doi:10.1186/s13643-020-01551-y)
Supplement: Supplementary file 2 — Additional file 2. Codebook. [file 13643_2020_1551_MOESM2_ESM.pdf]

The Influence of Acute Exercise on Bone Biomarkers: Protocol for a Systematic Review with Meta-Analysis

Supplementary File Two

Codebook

**Column**		**Heading**	**Description**
**STUDY DETAILS**	A	Study Number	Study number
	B	Author	First author surname *et al.*,
	C	Year	Year of publication
	D	Journal	Journal name
	E	Title	Study title
	F	Funding/COI	List all funding sources, and any declared conflict of interest.
	G	Aim	Study aim
	H	Design	Main study design, with brief description of conditions investigated.
	I	Design Code	Experimental Trials = 1; Observational Trials = 2
	J	Nutritional Intervention	1 = Studies without a nutritional intervention; 2 = Studies that include a nutritional intervention (e.g., exercise conducted with and without calcium supplementation)
	K	Nutritional Intervention	If column H is coded 1, provide a brief description of the nutritional intervention under investigation.
**POPULATION**	L	Participant Overview	Brief descriptive overview of the participant population (age, sex, health and training status)
	M	Starting n	Number of individuals initially enrolled in the study.
	N	End n	Number of individuals who finished the study.
	O	Training Status	Come up with coding categories.

	P	Sex	1 = male, 2 = female, 3 = mixed male and female group.
	Q	Age	Mean (yrs)
	R	Age	SD (yrs)
	S	Height	Mean (cm)
	T	Height	SD (cm)
	U	Weight	Mean (kg)
	V	Weight	SD (kg)
	W	BMI	Mean
	X	BMI	SD
	AB	Comments	Any additional information relevant information related to the participants investigated.
**EXERCISE TEST DETAILS**	AC	Exercise stimulus	Brief narrative description of the test undertaken.
	AD	Type	1 = resistance (defined as exercises that cause the body's muscles to work or hold against an applied force or weight, *e.g.*, weight lifting); 2 = aerobic (defined as activities whereby large muscle groups move in a rhythmic manner for a sustained period of time, *e.g.*, walking, running or cycling); 3 = multi-modal (defined as exercise bouts that comprise a combination of exercise modalities, *e.g.*, sessions that comprise a mixture of both resistance and aerobic exercises); 4 = plyometric (high-impact exercise types designed to develop muscular power, *e.g.*, jump based exercise bouts); 5 = calisthenics (systematic rhythmic body weight exercises, *e.g.*, yoga or pilates).
	AE	Aerobic type	1 = running; 2 = cycling; 3 = other.
	AF	Aerobic type	1 = continuous; 2 = intermittent.
	AG	Intensity	Effort related to maximum capacity, e.g., % VO_2 max or 1RM.
	AH	Duration	Minutes.
	AI	Total work done	Intensity*Duration

	AJ	Impact level	1 = low-impact/repetitive; 2 = moderate-impact/repetitive; 3 = low-impact with high muscular load; 4 = high-impact/multi-directional.
	AK	Exercise termination	1 = conducted to exhaustion; 2 = fixed load.
**SAMPLING**	AL	Samples	Brief narrative description of the number and timing of samples taken.
	AM	Baseline condition	Time of day
	AN	Baseline condition	1 = fed; 2 = fasted
	AO	Bone biomarkers	List all bone biomarkers assessed.
	AP	Other biomarkers	List all other biomarkers assessed.
	AQ	Bone biomarker	1 = bone specific alkaline phosphatase (B-ALP); 2 = dickkopf-1 (DKK-1); 3 = carboxyterminal propeptide of type 1 procollagen (P1CP); 4 = N-terminal propeptide of type 1 procollagen (P1NP); 5 = sclerostin; 6 = pyridinoline (Pyr); 7 = deoxypyridinoline (Dpd); 8 = carboxyterminal telopeptide of type-1 procollagen (ICTP); 9 = aminoterminal telopeptide of type 1 collagen (NTx); 10 = cathepsin K; 11 = C-terminal telopeptide of type 1 collagen (β-CTX-1); 12 = tartrate resistance acid phosphatase isoenzyme 5b (TRAP5b), 13 = OPG/RANKL ratio, 14 = hydroxylysine; 15 = hydroxyproline) 16 = osteopontin; 17 = osteocalcin; 18 = calcium; 19 = phosphorus; 20 = parathyroid hormone
	AR	Subtype	If information regarding specific biomarker subtype is provided, insert as free text.
	AS	Process	1 = Formation (bone specific alkaline phosphatase (B-ALP); dickkopf-1 (DKK-1); carboxyterminal propeptide of type 1 procollagen (P1CP) and N-terminal propeptide of type 1 procollagen (P1NP) and sclerostin); 2 = Resorption (pyridinoline (Pyr); deoxypyridinoline (Dpd); carboxyterminal telopeptide of type-1 procollagen (ICTP); aminoterminal telopeptide of type 1 collagen (NTx); cathepsin K; C-terminal telopeptide of type 1 collagen (β-CTX-1); tartrate resistance acid phosphatase isoenzyme 5b (TRAP5b), the ratio of osteoprotegerin to receptor activator NF kappaB ligand (OPG/RANKL), hydroxylysine and hydroxyproline); 3 = General (osteopontin and total and undercarboxylated osteocalcin (T/U-OC); 4 = Ca Metabolism (ionized or albumin adjusted calcium, phosphorus and parathyroid hormone)

	AT	Direction	1 = increase represents an increase in the relevant process; -1 = increase represents a decrease in the relevant process (e.g., sclerostin has an anti-formation action).
	AU	Sample type	1 = serum; 2 = plasma; 3 = urine.
	AV	Process	Brief narrative description of the assay used/assessment type.
	AX	Inter-assay variability	%
	AY	Intra-assay variability	%
	AZ	Unit	Unit of measurement
**MAIN DATA**	BA	Baseline	Mean
	BB	Baseline	SD
	BC	Time	Exact time at which the measurement was taken
	BD	Time	1 = sample taken immediately before the exercise bout (i.e., within 15 minutes before exercise commencement); 2 = samples taken within 15 minutes and 2 hours before exercise commencement); 3 = samples taken > 2 hours before exercise commencement)
	BE	Post Exercise	Mean
	BF	Post Exercise	SD
	BG	Time	Exact time at which the measurement was taken
	BH	Time	1 = sample taken immediately post exercise (i.e., within 15 minutes of exercise termination); 2 = samples taken within 15 minutes and 2 hours post exercise; 3 = samples taken within 2 and 5 hours post exercise; 4 = samples taken 1 day post exercise; 5 = samples taken 2 days post exercise; 6 = samples taken 3 days post exercise; 7 = samples taken 4 days post exercise, *etc*.
	BI	During/Post	1 = sample taken post exercise; 2 = sample taken during the exercise bout.

	BJ	Comment	Any other relevant comments
