# Supplementary material for: The influence of acute exercise on bone biomarkers: protocol for a systematic review with meta-analysis
Source: Syst Rev. 2020 Dec 12;9:291. doi: 10.1186/s13643-020-01551-y (PMC7733242; doi:10.1186/s13643-020-01551-y)
Supplement: Supplementary file 4 — Additional file 4. Modified Downs & Black checklist. [file 13643_2020_1551_MOESM4_ESM.pdf]

The Influence of Acute Exercise on Bone Biomarkers: Protocol for a Systematic Review with Meta-Analysis

Supplementary File 4

Modified Downs & Black checklist.

Q.1. *Is the hypothesis/aim/objective of the study clearly described*? (Yes = 1; No = 0)

Q.2. *Are the main outcomes to be measured clearly described in the introduction or methods section?* If the main outcomes are first mentioned in the results section, answer no. (Yes = 1; No = 0).

Q.3. *Are the characteristics (e.g., age, height, weight, training and health status) of the participants included in the study clearly described?* (Yes = 1; No = 0).

Q.4. *Are the interventions of interest clearly described?* For exercise interventions, the type, intensity and duration should be described. If they provide a nutritional supplement the exact type and dose should be provided (Yes = 1, No = 0).

Q.5. *Are the main findings of the study clearly described?* Simple outcome data should be reported for all major findings so the reader can check the major analyses and conclusions. This does not cover statistical tests. (Yes = 1; No = 0).

Q.6. *Does the study provide estimates of the random variability in the data for the main outcomes?* In non-normal data, inter-quartile range should be reported. In normal data, standard deviation, standard error or confidence intervals should be reported. (Yes = 1; No = 0).

Q.7. *Have all important adverse events that may be a consequence of the intervention been reported*. Answer yes if they confirm they have ethical approval (Yes = 1; No = 0).

Q.8. *Was an attempt made to blind study subjects to the intervention they have received?* For exercise interventions where it is not possible to blind, answer yes. Answer no if the intervention contains a supplement/placebo arm, and this is not blinded. (Yes = 1, no = 0, unable to determine = 0)

Q.9. *Was an attempt made to blind those measuring the main outcomes of the intervention?* For exercise interventions where it is not possible to blind, answer yes. Answer no if the intervention contains a supplement/placebo arm, and this is not blinded. (Yes = 1, no = 0, unable to determine = 0)

Q.10. *If any of the results of the study were based on 'data dredging' was this made clear?* Any analyses that had not been planned at the outset should be clearly indicated. If no retrospective subgroup analyses were reported, then answer yes. (Yes = 1; No = 0; Unable to determine = 0).

Q.11. *Was the timing of blood sampling clearly described?* Answer yes if the precise time-points were provided. Answer no if it is not clear exactly when the blood samples were drawn (Yes = 1, no = 0, no = 0).

Q.12. *Were the statistical tests used to assess the main outcomes appropriate?* (Yes = 1, no = 0, Unable to determine = 0).

Q.13. *Were the main outcome measures used accurate (valid and reliable)?* For studies where the outcome measures are clearly described, the question should be answered yes. For studies which refer to other work that demonstrates the outcome measures are accurate, or that use commonly used tests, the question should be answered yes (Yes = 1, No = 0; Unable to determine = 0).

Q.14. *Were study subjects randomised to intervention groups?* Answer yes if the order of treatment, or allocation to groups, was randomly assigned. If it was not possible for the study to be randomised (e.g., single-trial studies) answer yes. (Yes = 1; No = 0; Unable to determine = 0)

Q.15. *Was at least one familiarization session conducted prior to exercise testing?* Answer yes if they conducted a familiarization trial, or if familiarization was not necessary (*e.g.,* if the study uses a single, non-performance-based, exercise bout). (Yes = 1; No = 0; Unable to determine = 0).

Q.16. *Were the exercise test conditions adequately standardised and described?* Factors to consider include confirmation of the time of day that testing was conducted, and control for unusual activity or nutritional factors in the days prior to the exercise test. Yes (all 3 factors considered) = 3; Yes (most

factors (2 of the 3) considered) = 2; Yes (some (1 of the 3) factors considered) = 1; No = 0; Unable to determine = 0.

Q.17. *Was nutritional status for blood sampling adequately described*? Answer yes if the strategy for standardization was described in sufficient detail to allow for replication. (Yes = 1, No = 0, Unable to determine = 0)

Max attainable score = 19. The combined score will be used to categorise each study according to 4 categories, i.e., High (17 - 19), Moderate (14 - 16), Low (7 – 11 - 13) or Very Low (< 10)

Original Reference:

Downs SG & Black N. The feasibility of creating a checklist for the assessment of the methodological quality both of randomised and non-randomised studies of health care interventions. *J Epidemiol Community Health*. 1998; 52(6): 377 – 384.
