# Supplementary material for: The influence of acute exercise on bone biomarkers: protocol for a systematic review with meta-analysis
Source: Syst Rev. 2020 Dec 12;9:291. doi: 10.1186/s13643-020-01551-y (PMC7733242; doi:10.1186/s13643-020-01551-y)
Supplement: Supplementary file 3 — Additional file 3. PRESS Guideline — Search Submission & Peer Review Assessment. [file 13643_2020_1551_MOESM3_ESM.pdf]

PRESS Guideline — Search Submission & Peer Review Assessment

SEARCH SUBMISSION: THIS SECTION TO BE FILLED IN BY THE SEARCHER

Searcher: Eimear Dolan **E-mail:** eimeardol@gmail.com

Date Submitted: **Date Requested By:**

Systematic Review Title: The Influence of Acute Exercise on Bone Biomarkers: Protocol for a systematic review and meta-analysis

This search strategy is …

**X**	My PRIMARY (core) database strategy — First time submitting a strategy for search question and database
	My PRIMARY (core) strategy — Follow-up review NOT the first time submitting a strategy for search question and database. If this is a response to peer review, itemize the changes made to the review suggestions
	SECONDARY search strategy— First time submitting a strategy for search question and database
	SECONDARY search strategy — NOT the first time submitting a strategy for search question and database. If this is a response to peer review, itemize the changes made to the review suggestions

Database

Medline

Interface

OVID

Research Question

The aim of this investigation is to use the systematic review and meta-analytic approach to quantify the effect of an acute bout of exercise on circulating biomarkers indicative of bone metabolism, as well as investigate potential factors that may moderate this response, *e.g.*, variation in participant, exercise and sampling characteristics.

**Table 1.** Overview of PICOS eligibility criteria.	
Participants	In order to allow for analysis of whether participant characteristics (sex, age, health or training status) will impact the bone biomarker response to a single bout of exercise, men and women of any age, health or training status will be considered for inclusion in this review.
Intervention	Studies that investigate a single exercise bout of any duration, intensity or type will be considered for inclusion.
Comparator	The primary comparison of interest is the pre – post difference in bone biomarkers as a result of an acute exercise bout.
Outcomes	The primary outcome of interest is the bone biomarker response to an acute exercise bout. Bone biomarkers will be considered in relation to the processes of **bone formation** (bone specific alkaline phosphatase (B-ALP); dickkopf-1 (DKK-1); carboxyterminal propeptide of type 1 procollagen (P1CP) and N-terminal propeptide of type 1 procollagen (P1NP) and sclerostin), **bone resorption** (pyridinoline (Pyr); deoxypyridinoline (Dpd); carboxyterminal telopeptide of type-1 procollagen (ICTP); aminoterminal telopeptide of type 1 collagen (NTx); cathepsin K; C-terminal telopeptide of type 1 collagen (β-CTX-1); tartrate resistance acid phosphatase isoenzyme 5b (TRAP5b), the ratio of osteoprotegerin to receptor activator NF kappaB ligand (OPG/RANKL), hydroxylysine and hydroxyproline, and general **bone remodelling** (osteopontin and total and undercarboxylated osteocalcin (T/U-OC).

Study Designs	Any study design that includes measurement of bone biomarkers before and after an acute, bout of exercise will be considered for inclusion. These include randomized and non-randomized controlled trials, including cross-over trials, as well as single group pre-post studies. Our rationale for including these different designs is based on 1) our desire to be as inclusive as possible, 2) to identify if results are associated with these different designs, and 3) provide direction for future research with respect to designing studies aimed at examining the effects of an acute bout of exercise on bone biomarkers. Natural experiments, *i.e.,* studies that measure bone biomarkers before and after real-life athletic events (e.g., endurance events) will also be considered for inclusion, however because these investigations are prone to substantially more variation than experimental laboratory studies, they will not be included in the main analysis and instead a secondary analysis will be undertaken using these data.

Was a search filter applied?

Yes X No ■

Studies were filtered to include human studies only.

Please copy and paste your search strategy here, exactly as run, including the number of hits per line. ***[mandatory]***

Preliminary Search Strategy for the Medline Database

Ovid®

My Account | Support & Training | Contact your Library Service | Help

Search | Journals | Books | Multimedia | My Workspace | What's New

▼ Search History (12)

# ▲	Searches	Results	Type	Actions
1	bone.mp. or "Bone and Bones"/	957226	Advanced	Display Results | More ▾
2	Exercise/ or exercise.mp.	359857	Advanced	Display Results | More ▾
3	physical activity.mp.	114381	Advanced	Display Results | More ▾
4	biomarkers.mp. or Biomarkers/	539836	Advanced	Display Results | More ▾
5	turnover.mp.	101121	Advanced	Display Results | More ▾
6	Bone Remodeling/ or remodeling.mp.	125194	Advanced	Display Results | More ▾
7	formation.mp.	1155930	Advanced	Display Results | More ▾
8	Bone Resorption/ or resorption.mp.	62019	Advanced	Display Results | More ▾
9	2 or 3	418547	Advanced	Display Results | More ▾
10	4 or 5 or 6 or 7 or 8	1896333	Advanced	Display Results | More ▾
11	1 and 9 and 10	2373	Advanced	Display Results | More ▾
12	limit 11 to humans	1709	Advanced	Display Results | More ▾

Date Conducted: November 21, 2020. Papers retrieved, n = 1709

**Overview**	
User Query	(bone) AND (exercise OR physical activity) AND (biomarkers OR turnover OR remodelling OR formation OR resorption)<br><br>A combination of free text and database specific subject headings will be used. The OVID advanced search multi-purpose (mp) function will be used.
**Breakdown**	
Population	NA
Intervention	(exercise OR physical activity)
Outcome	(bone) AND (biomarkers OR turnover OR remodeling OR formation OR resorption)

PEER REVIEW ASSESSMENT: THIS SECTION TO BE FILLED IN BY THE REVIEWER

Reviewer:	Email:	Date completed:

1. TRANSLATION

A ---No revisions	☐
B --- Revision(s) suggested	☐
C --- Revision(s) required	☐

If "B" or "C," please provide an explanation or example:

2. BOOLEAN AND PROXIMITY OPERATORS

A ---No revisions	☐
B --- Revision(s) suggested	☐
C --- Revision(s) required	☐

If "B" or "C," please provide an explanation or example:

3. SUBJECT HEADINGS

A ---No revisions	☐
B --- Revision(s) suggested	☐
C --- Revision(s) required	☐

If "B" or "C," please provide an explanation or example:

4. TEXT WORD SEARCHING

A ---No revisions	☐
B --- Revision(s)suggested	☐
C --- Revision(s) required	☐

If "B" or "C," please provide an explanation or example:

5. SPELLING, SYNTAX, AND LINE NUMBERS

A ---No revisions	☐
B --- Revision(s)suggested	☐
C --- Revision(s) required	☐

If "B" or "C," please provide an explanation or example:

6. LIMITS AND FILTERS

A ---No revisions	☐
B --- Revision(s) suggested	☐
C --- Revision(s) required	☐

If "B" or "C," please provide an explanation or example:

OVERALL EVALUATION (Note: If one or more "revision required" is noted above, the response below must be "revisions required".)

A ---No revisions	☐
B --- Revision(s) suggested	☐
C --- Revision(s) required	☐

Additional comments:
